# Gender differences in traditional knowledge of useful plants in a Brazilian community

**DOI:** 10.1371/journal.pone.0253820

**Published:** 2021-07-28

**Authors:** Fernanda Vieira da Costa, Mariana Fernandes Monteiro Guimarães, Maria Cristina Teixeira Braga Messias

**Affiliations:** Departamento de Biodiversidade, Programa de Pós-graduação em Ecologia de Biomas Tropicais, Evolução e Meio Ambiente, Universidade Federal de Ouro Preto, Ouro Preto, Minas Gerais, Brazil; Universidade Federal de Pernambuco, BRAZIL

## Abstract

Genders differ in traditional ecological knowledge (TEK) about plants, but how gender influences TEK sharing is still poorly understood. Here, we examined how gender is associated with the diversity, transmission, and structure of TEK. We tested whether women and men differ in terms of plant knowledge (species richness, α-diversity), knowledge heterogeneity (β-diversity), and in the structure of social-ecological networks they form. The study was carried out in a suburban community in the city of Ouro Preto, Southeastern, Brazil. Using the snow-ball technique, semi-structured interviews, guided tours, and participant observation, we gathered information from 33 women and 33 men in the community. We collected information about their culture, social-economic profiles, and plant knowledge from which we identified 291 plant species in 10 use categories. Overall, our results indicated that the cognition and sharing of ethnobotanical knowledge are structured by gender. Women rated better in their plant knowledge repertory (greater α-diversity), while plant knowledge among men was more heterogeneous (greater β-diversity), suggesting less information sharing among them. We observed that the network among women is more connected, exhibited greater information sharing, with a greater number of central individuals, who likely provide the cohesion and maintenance of TEK in the community. Our findings indicate how social-ecological networks can provide insights and information to unveil social patterns of knowledge transmission. Understanding how TEK is fostered and shared among community members will favor better planning of ethnobotanical studies, as well as inform decision-makers about strategies for the conservation of plant TEK.

## Introduction

Knowledge and use of plants by traditional communities are products of experimentation and information exchange among those who share the culture and beliefs in resource use in a given place [1,2], often called the social-ecological systems of traditional knowledge [3,4]. Two processes are fundamental in the structure of social-ecological systems: Knowledge gathered through individual production and knowledge shared within the community [5,6]. Many social and ecological aspects involved in those two processes within these traditional systems are not yet understood, and continued research is required [3,4].

Oral transmission of traditional knowledge is the most common way that this knowledge is passed on. The ability of learning with others favors the process of information acquisition without the experimentation cost. Nonetheless, new experimentation can be used as a way to improve on the knowledge already obtained [7]. The most common form of traditional knowledge transmission is the vertical one, which occurs between generations of a family [8]. Several important scientific works have brought strong evidence that social-cultural systems where the pattern of transmission is determined mainly by vertical transmission exhibit greater heterogeneity of knowledge among members of the community, because each family has its own specialties and experiences [5–7]. However, the extent of vertical transmission in ethnobotanical studies is often overestimated, and so it may appear more important than it really is, especially when reported as a response to questions in interviews [9]. Horizontal transmission (among individuals in the same generation but not necessarily in the same family) and oblique transmission (among non-family members of different generations) are also important knowledge transmission strategies [6,10], and they tend to result in more homogeneous knowledge [2,11]. Despite this evidence, the relationship between knowledge heterogeneity (i.e., knowledge variation) and its transmission is not trivial and still poorly explored.

To better understand the dynamics of how traditional ecological knowledge (TEK) and associated practices are passed on to others in the community, we must also understand community dynamics and how people, their roles, and their ideas are organized and interact over time and space [11,12]. It is known that the division of labor in communities can vary with scale of survey [12,13] and thus, determines variations in the way people perceive and use plant resources [1,14,15]. Thus, social organization affecting the division of labor needs to be examined to better understand TEK transmission and diversity patterns [1]. Cultural, social and economic factors (such as age, gender, education and income) also influence the transmission of knowledge, and consequently, social-ecological systems of knowledge [13,15,16].

Gender is clearly among the key factors in structuring social-ecological systems of knowledge [14,17]. The knowledge difference between genders can vary with the scale of observation (e.g., national, continental, or global) and some authors argue that differences on knowledge richness are easily to find on smaller scales [12]. Thus, communities with well-defined gender roles tend to have greater gender differences in TEK [12,17,18]. Many ethnobotanical studies have pointed out that women are inclined to know more about medicinal and food plants used within the household, as part of subsistence living and family care with plants usually found more locally, such as homegardens and other habitats around their houses [12,19–22]. On the other hand, studies attribute to men a greater knowledge and use of timber plants, usually for construction or commercial purposes, which may be widely dispersed over the landscape and more distant from the residence place [20–22]. Studies of gender roles in ethnobotany usually have focused simply on the species richness in different plant-use categories recognized by each gender [1,23], so much remains to be unveiled about gender roles in maintaining and sharing ethnobotanical knowledge. Gender differences in social interactions are reasonably well-known, and illustrated in some important technical books [16,24], as well in a variety of scientific studies (e.g., [1,11–14,17,21,25,26]). However, while some aspects of TEK are understood, we are a long way from understanding gender roles in ethnobotany, where each sex may have different specialties or focus on the variety of plants that are used by the community [1,17,27,28].

Tools borrowed from community ecology [29] can be easily adapted in studies that aim to assess gender roles in local plant knowledge and its transmission. Using estimates of α-diversity (in ethnobotany meaning the richness of known useful plants), β-diversity (as the variability in known useful plant species among individuals), gamma diversity (as the total species richness of useful plants recognized by the community), and ecological networks (as the structural organization of social-ecological systems), we can compare patterns of social-ecological knowledge between genders. Thus, by assessing the gamma diversity in the entire community, we can disentangle the α-diversity (i.e., the repertory of known plants) and the β-diversity (typically used by ecologists to explain spatial or temporal diversity, e.g., [30]) that each gender holds, determining how heterogeneous and shared is this knowledge by each group. Recent findings have shown that more connected animal-plant ecological networks indicate greater resource sharing [31], so that we may expect that more connected social-ecological networks have greater knowledge sharing. In spite of not assessing diversity metrics, it was noted that the women presented a more homogeneous repertory of known species due to the higher socialization of knowledge between them [11]. Additionally, analogous to central species in ecological networks [31,32], central individuals in social-ecological networks may be important sources of knowledge acquisition and sharing (cultural transmission), and therefore are important for maintaining the structure and persistence of TEK, contributing to the resilience of the social-ecological systems [11].

Since the diversity and sharing of TEK are important attributes to define the patterns of social-ecological systems [3,4,16], here we examined the importance of gender in structuring the diversity of plant knowledge. Additionally, we examined gender influence in plant TEK transmission, testing whether gender is associated with differences in the social-ecological network structure. We attempt to answer the following questions: 1) Does the richness of known useful plants (α-diversity) depend on gender influence? 2) Are there gender differences in the categories of known useful plants? 3) Does the heterogeneity of useful plant knowledge (β-diversity) differ between genders? 4) Are there gender differences in how knowledge of useful plant species is structured and shared?.

Traditionally, women are recognized as housekeepers with greater knowledge of medicinal and edible plants in their repertoires [33]. Therefore, we predicted that women are familiar with more species and their uses than men [21,33]. In addition, as women share more information with each other than men [11,26], we hypothesized that their repertoire of known species is more homogeneous (lower β-diversity) than that of the men, i.e., we predicted a greater knowledge and homogeneity among women. Furthermore, we expected that among women, the TEK network will have higher connectance, lower isolated groups (i.e., lower modularity), higher sharing (i.e., higher overlap in known plants), and more central individuals that are more closely connected. In contrast, the network among men is expected to have fewer central individuals and fewer connections among other individuals–i.e., less connectance, more isolated groups and lower sharing). In these lines, central individuals in social-ecological networks should be those that are “repositories” of knowledge and therefore are also important for knowledge transmission within the community.

## Materials and methods

### Study area

**Fig 1:**
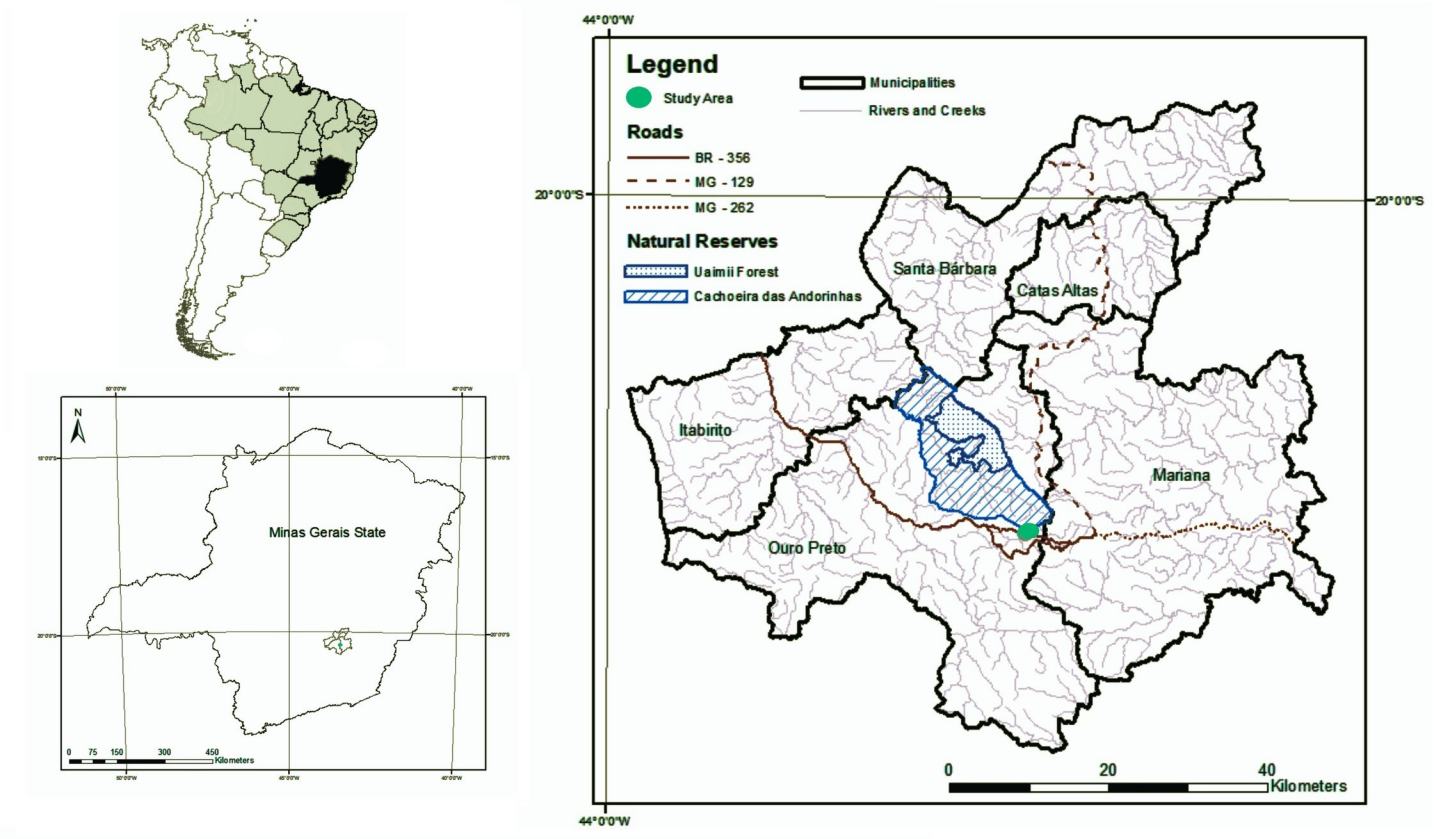
Location of the study area in the municipality of Ouro Preto, Minas Gerais State, Brazil.

Our case study was carried out in a community located at the northeast of the urban zone of Ouro Preto municipality, in the state of Minas Gerais, Brazil (20°30’ S, 44°33’ W, Fig 1). Ouro Preto has been recognized as World Heritage Site [34] and in Brazil, is recognized for its national heritage, part of which includes popular knowledge of medicinal plants [35]. Local plant knowledge has come from a variety of sources, in part due to the multi-ethnic nature of the people in the region, including indigenous peoples, African and Europeans that have mixed since colonization [35]. Urbanization in the region began towards the end of the 1700s during the colonial period with the discovery of gold [34]. Today, the municipality has around 74,000 inhabitants in 1,245,865 km^2^, and the studied community comprises less than 5% of the city population [36]. The income of the studied community is lower than the average income of the municipality. Residents of the Ouro Preto suburban areas, like those of the study area use more native plant species than those who live in the city center [35].

The local vegetation is within the Atlantic Forest and the Cerrado (Brazilian savannas) domain, which locally is mountainous, comprising mostly by *campos rupestres* (Brazilian rock outcrops) and seasonal semideciduous forests [37]. Climate is humid mesothermic (Cwb in the Köppen scheme), with a warm rainy season from November to March, and a drier cold season [38]. Annual rainfall is about 1,250 mm and average annual temperature is 20°C. Several areas protected for conservation can be found around the urban center of Ouro Preto. We studied a suburban community adjacent to two of those protected areas (Uamií State Forest and Cachoeira das Andorinhas Park, Fig 1) where people have been managing plant resources for some time [39].

### Sampling methods

After approval by the Research Ethics Committee (CEP) of the Federal University of Ouro Preto (CAAE: 05301712.4.1001.5150) and obtaining the license to collect botanical material (IEF No. 122/2014 and 002/2015), we collected the ethnobotanical data from November 2014 to June 2016. We first presented our project to the community during a round-table discussion, when we introduced ourselves and our objectives. Interviewees were selected by the snowball technique, and the initial ones were indicated in the meeting to present the project to the community. The snowball technique is a non-probabilistic technique [40,41], which helps to identify a “network of experts”, where interviewees indicate the next ones until they begin to repeat, indicating the sampling saturation point [42]. As inclusion criteria, only residents older than 18 years who lived in the community for at least 10 years were selected. These people self-identified as persons knowledgeable about plants and accepted our invitation to participate in this study. All participants signed the terms of clear and free consent in conformance with the Brazilian resolution (No. 196/96).

We used semi-structured interviews, in which a list of pre-determined questions was organized into a script that allowed flexibility during the interview [16]. In this way, we gathered information about participant social, economic and historical situations (gender, age, income, and education level), as well as what they knew about the environment, the plants they used (plant names, plant use, plant parts they used, etc., see S1 Table for detailed information) and the way of plant knowledge acquisition (by vertical/parental, horizontal and oblique paths). Interviews were scheduled and then carried out at the house of the participants, maintaining a friendly, respectful and open attitude that allowed the participant to freely contribute with any additional information they thought relevant. In addition to interviews, we also used participant observation [16], in which we obtained extra information on knowledge transmission inside the community. It is important to mention that the researchers have lived with the local community for more than 10 years, an experience that enables us to perceive and analyze the community´s observed reality, ‘from within’, with an emic view. These observations encompass, among other aspects, the culture, people relationship (including TEK transmission), and people-plant relationship [16].

We also went on guided tours [16] with the participants in the regions in which they collect the plants, periods when they showed us some of the plants and we continued with informal questions that complemented the other information already provided. During these guided tours we collected plant samples that we placed in the reference collection at the Professor José Badini Herbarium (OUPR) of the Federal University of Ouro Preto (UFOP), following typical herbarium methods [43]. Specimens were identified by typical botanical methods, including morphological analysis using taxonomic keys and literature, comparisons with herbarium specimens, and consultation with taxonomists, as necessary. Families followed APG III [44] and the species nomenclature followed The Plant List database [45].

### Data analysis

Social-ecological variables were subdivided into categories, including gender (female, male); age (< 25 years, 26–35, 36–45, 46–55, 56–65, 66–75, and > 75), education (illiterate, incomplete elementary school, complete elementary school, incomplete high school, complete high school, incomplete higher education, and complete higher education); and income (measured as Brazilian minimum-salaries, thus, < 1, 1–2, 2.1–3, 3.1–4, 4.1–5, 5.1–6 and > 6). We compared the frequencies of other social-economic variables (age, education, and income) between gender groups using the G-test [46] in order to verify any other difference able to influence the response despite gender. Plant uses were grouped into categories (cosmetic, ecological, edible, fodder, fuel, medicinal, mystic, ornamental, technological, and timber), as proposed by Albuquerque et al. [16]. We also compared genders in the frequencies of plant knowledge by different use categories using the G-test [46].

To test whether the richness of known plants differ between women and men, we calculated the alpha (*α*) diversity of known species, which corresponds to the total number of species cited by each individual. The beta (*β*) diversity of known plants was calculated using the multiplicative partitioning approach proposed by Whittaker [47]: $\beta =\frac{\gamma }{\alpha }$, where the gamma diversity (*γ*) corresponds to the total number of known species cited by men and women. Thus, to test whether *α* and *β*-diversity depend on gender influence, we built Generalized Linear Models (GLM), wherein *α* and *β*-diversity were response variables and gender was the predictor variable.

To test whether the structure of socio-ecological networks differ between genders we used binary matrices in which participant *i* identified or used plant species *j*, from which we calculated plant networks by gender. We then examined how network structure was influenced by gender using the following metrics widely used in ecological network studies: frequency of interactions, connectance, niche overlap in resource use, and modularity (e.g., [30,31]). Higher connectance (values near one) indicate greater cohesion among individuals. Lower values of niche overlap (Morisita-Horn index, values from 0 to 1) indicate lower resource sharing among participants, i.e., uniqueness of knowledge, while higher values indicate greater resource sharing. Greater modularity (QuanBiMo algorithm, in which Q-values vary from 0–1) indicates cohesive groups that are more connected in their botanical knowledge among themselves than with the remaining network [48]. We used the Patefield null model to estimate the probability of the observed network metrics based on 999 randomized networks [49]. All network metrics were calculated using bipartite package [50] in R [51].

To explore the importance of each participant in acquiring and sharing knowledge in social-ecological networks, we used three important centrality metrics for each individual: degree of centrality, closeness centrality, and betweenness centrality [32]. Degree of centrality indicates the number of interactions of each participant in the network [31,32] (here, the number of known plants). Closeness measures how close one participant is to the other participants in the same network [32], i.e., measured as the length of the smallest number of links that connect two individuals in the network. Greater closeness occurs when a plant species recognized by the participant is also recognized by many other individuals in the same network. Low closeness indicates unique knowledge. Betweenness centrality is the importance of a participant as a connector among distinct parts of the network. That is, the participant with large values of betweenness is connected to many other participants in the network [32], and as such, is an important individual in terms of knowledge sharing. Individuals with above-average centrality values (for the three metrics) were considered central and thus, are key-individuals for network cohesion and structure (see [30–32] for an ecological perspective).

All statistical analysis were performed in R [51] and network illustrations in the program Pajek [52].

## Results

We interviewed 66 individuals equally divided by gender (33 each) and all social-economic characteristics were similar between women and men. Age varied from 22–87 (mean—57 years) and the frequency of participants in distinct ages did not differ between genders (G = 6.55, df = 6, *p* = 0.4769). Likewise, the frequency of participants in distinct education levels did not change with gender (G = 8.29, df = 6, *p* = 0.2174), wherein most women and men only had elementary school education. The income of participants also was similar between genders (G = 11.87, df = 6, *p* = 0.1049), varying from one to six minimum salaries, with most participants receiving a single minimum salary.

Traditional knowledge of plants was reported in the interviews as gathered mostly through vertical transmission between generations (100% of participants), especially parent–offspring. Horizontal transmission was also often mentioned (61% among men, 73% among women, G = 0.66, df = 3, *p* = 0.719), and usually occurred between friends and members of the same sex (95% among men, 100% among women), and occasionally between spouses (3%). Oblique transmission of knowledge was mostly described as occurring among friends of the same gender (10% for both genders). Other forms of knowledge acquisition (internet, books, etc.) were relatively uncommon (6%, both genders).

A total of 291 plant species, in 86 families and 10 usage categories were cited by the participants (S1 Table). The most species-rich families were Asteraceae (34 species), Lamiaceae (22), Fabaceae (22), Solanaceae (17), Rosaceae (10), and Myrtaceae (8), while 45 families had only one species. Usage categories included medicinal, edible, ornamental, fodder, fuel, cosmetic, ecological, mystic, timber, and technological (S1 Table). Medicinal use had the greatest species richness (121), followed by edible (90), and ornamental use (25) (S1 Table).

**Fig 2:**
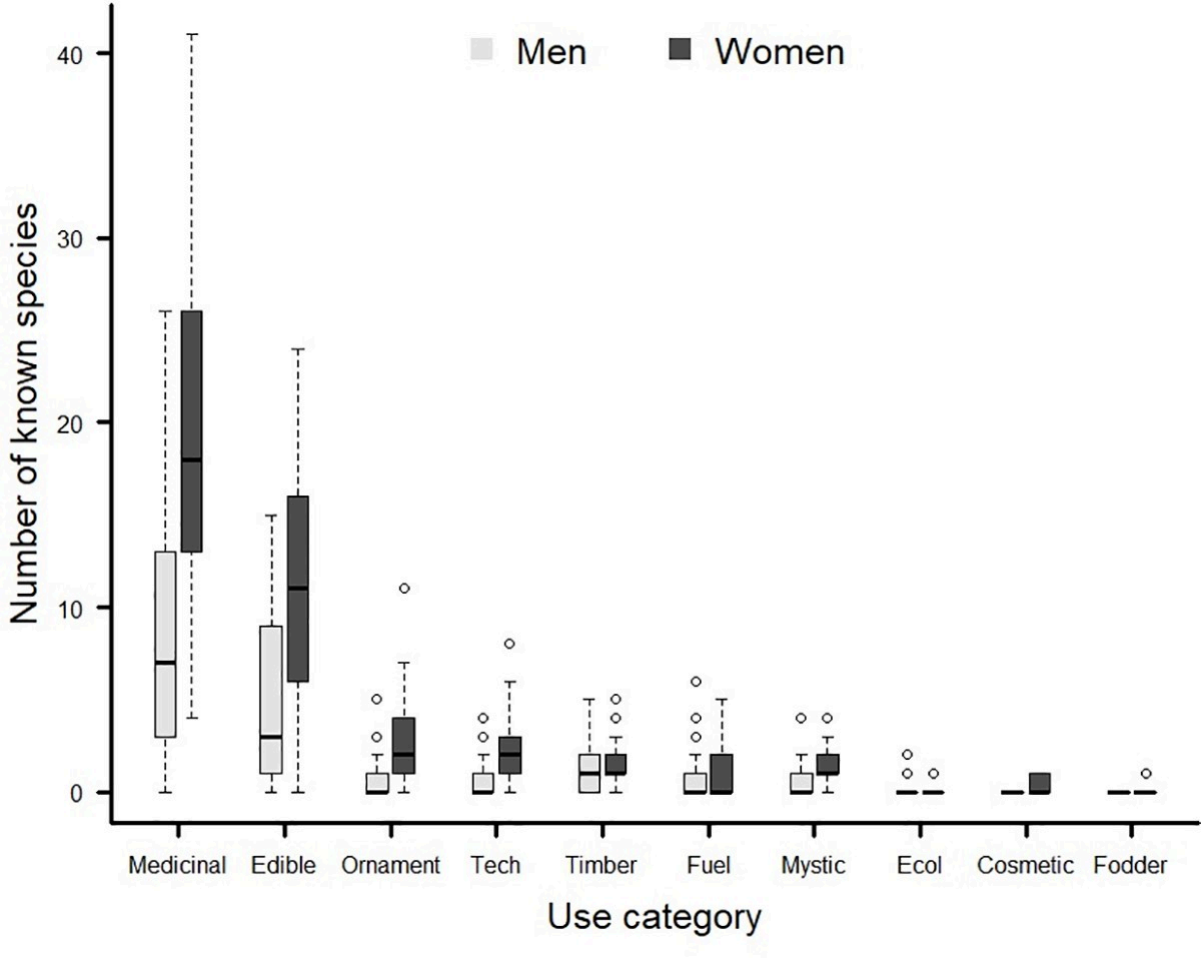
Number of known plant species in use categories by women and men in an urban community in Ouro Preto, in the state of Minas Gerais, southeastern Brazil.

**Fig 3:**
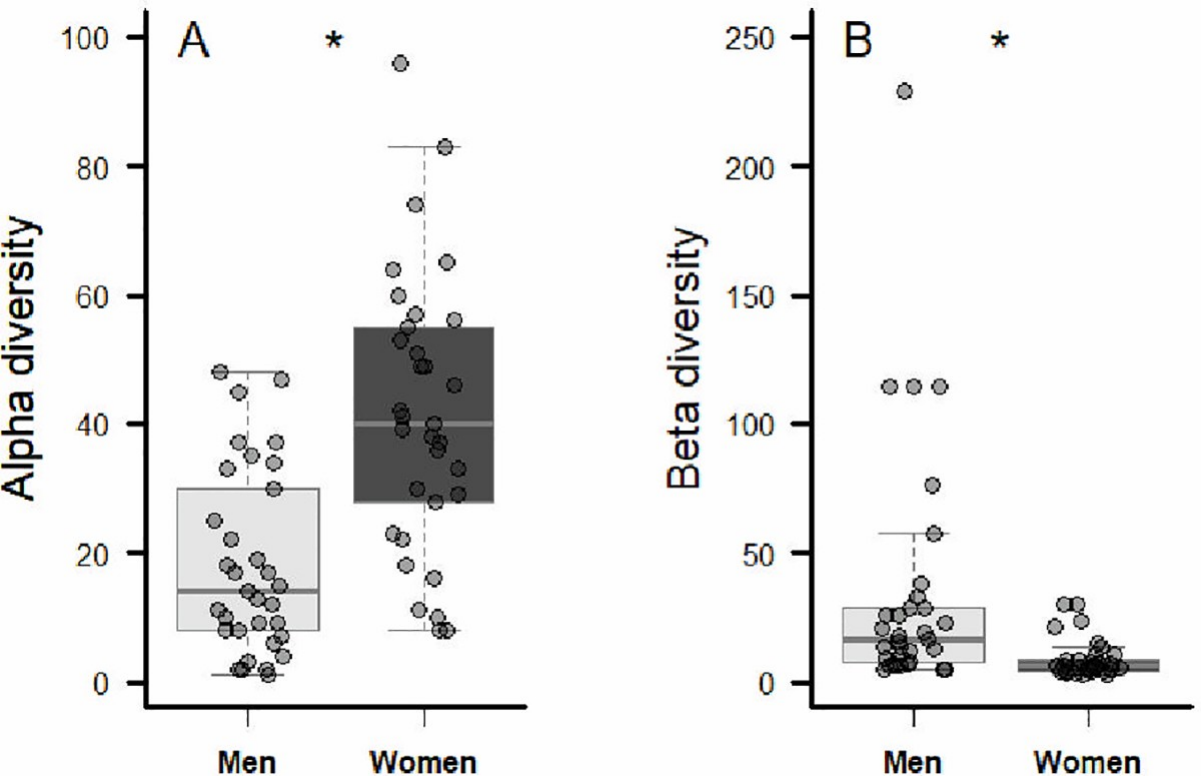
Comparisons between genders of alpha (α) and beta (β) diversity of known useful plants in an urban community in Ouro Preto, southeastern Brazil.

Both genders recognized plants by category in more or less the same frequencies (G = 2.57; df = 9; *p* = 0.98), with medicinal and edible the most common categories (Fig 2). Nevertheless, as expected, the number of known species (α-diversity) was higher among women (41.58 ± 21.4; Mean ± SD) than among men (18.18 ±14.2; Mean ± SD) (GLM: df-residual = 64, Deviance = 22.669, *p* < 0.0001; Fig 3A). The β-diversity though, i.e., the heterogeneity in plant-knowledge, was greater among men than women (GLM: df-residual = 64, Deviance = 10635, *p* = 0.003; Fig 3B).

**Fig 4:**
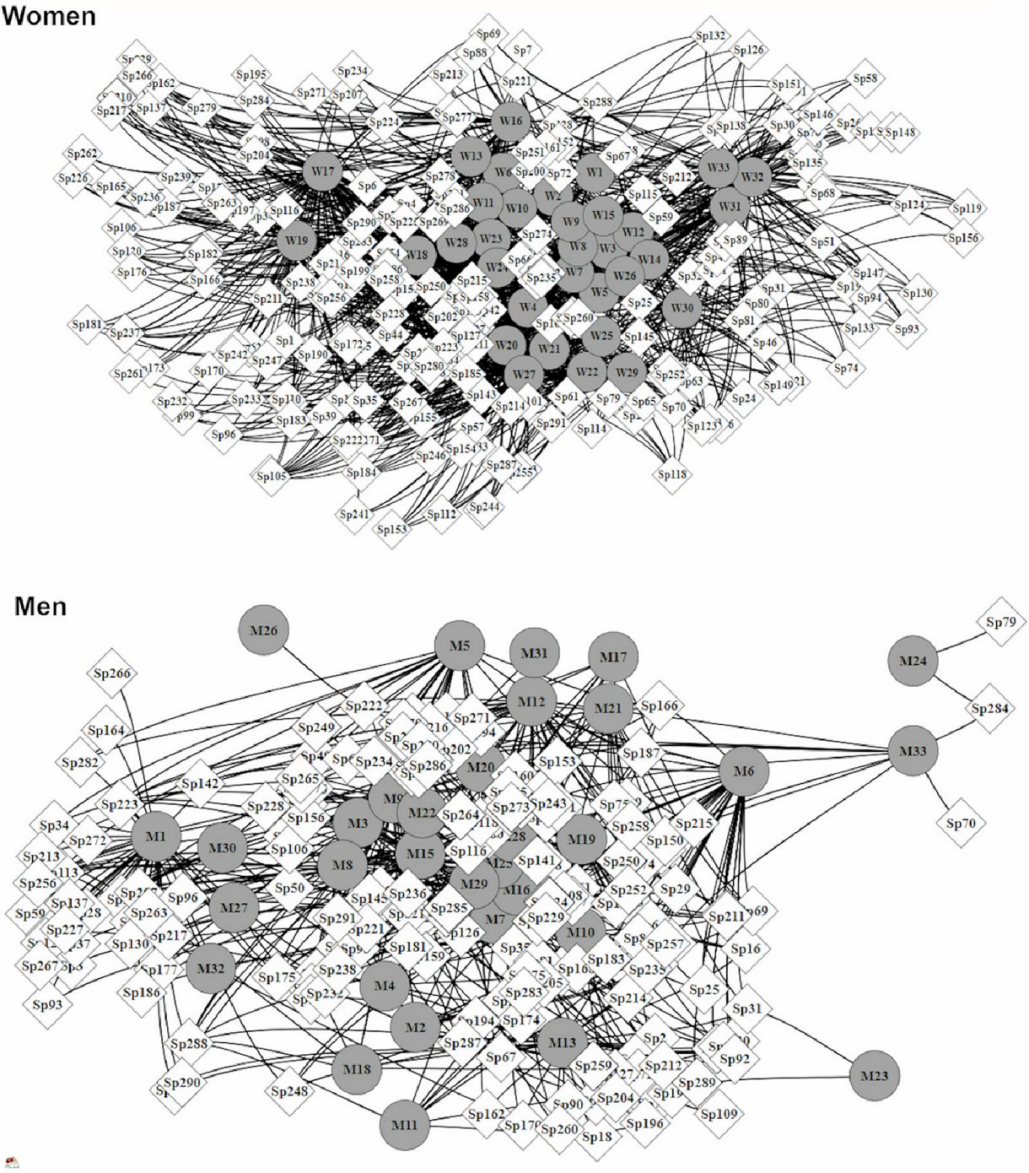
Socio-ecological networks among women and men illustrating the structure of traditional ecological knowledge on useful plants. Circles indicate participants (33 women and 33 men), while diamonds indicate each known plant species (n = 291). For plant species names see S1 Table.

For women, the observed network had 1372 interactions frequency, with 238 known plant species, while the network for men had less than a half of the frequency observed among women (600 records and 229 known species) (Fig 4). As predicted, the network among women was more connected (Connectance = 0.17 for women and 0.07 for men), had higher niche overlap in known plants (Horn = 0.21 and 0.07), and lower modularity (Q-value = 0.27 and 0.40, S2 Table). Additionally, 31 out of the 33 women participants (~94%) were central in terms of closeness and betweenness centrality, while 15 were central in terms of centrality degree (45%). In contrast, only 19 men (58%) were central by closeness, 13 (39%) by betweenness, and 12 by centrality degree (36%).

## Discussion

Our findings evidence that the knowledge of useful plants is structured by gender. We found that women have a greater repertory of known plant species (greater α-diversity) and tend to share what they know (lower β-diversity) more than men. Additionally, women, have a more cohesive network with more central individuals that make their network more cohesive. Our results disentangle some aspects of how gender is involved in horizontal sharing (acquisition/transmission) of traditional ecological knowledge (TEK). After discussing these main findings, we bring to light possible influences of gender in structuring social communication about useful plants, which ultimately affect the social-ecological network in traditional communities.

Despite gender differences in TEK, their repertories of known plants had similar patterns. The families Asteraceae, Fabaceae, and Lamiaceae were the most well-known plant families for both genders, a pattern already observed in other surveys within the studied region (mostly *campo rupestre* vegetation) [35,53,54], within the Atlantic Forest and Cerrado domains [55–57], and in other Latin American countries [19], where these families are all quite speciose [45]. In fact, these plant families present many species that have been cultivated worldwide, mainly by their medicinal and edible properties, which may explain their great species richness in ethnobotanical studies [58].

Our data strongly supported our predictions that women recognize more useful plant species than men, and that women’s knowledge is more homogeneous. Historically, women have been considered to be important repositories of plant knowledge, and considered to play an important role in the maintenance of knowledge on the use of plant resources [1,12]. Evidence suggests that greater ethnobotanical knowledge of women comes from their better ability to identify plants [1], especially those used for home care (e.g., edible, medicinal, ornamental), that are often collected on managed areas closer to home [1,19–21,33,35,59,60]. Furthermore, some studies have shown that women frequently manage plants from distinct environments independently of the landscape type (natural or anthropic) [1,59] and tend to better share their knowledge than men do [11]. However, a recent study pointed out that gender influence on the richness of known medicinal species is not a global pattern, only occurring on small scales, and that both men and women can stand out as more knowledgeable [12].

Surprisingly, while differences in the number of known plants and network structure were evident when comparing genders, the frequency of plants recognized by their use categories was similar between men and women. Other studies, in contrast, suggest that use categories generate differences in the types and richness of species used for distinct purposes, when comparing genders. For example, plants used for timber often fall within the purview of men, who will use the wood for construction. Women, on the other hand, being caregivers in the family and community, typically know more about the medicinal and edible plants [1,33,54,61]. However, in patriarchal systems such as those in Latin America, men usually fill the role of providing income for the family. Thus, the fact that women know about and manage plant resources for marketing purpose is often hidden or ignored [19]. Feminism, however, is gaining importance among women in Latin America, especially in urban communities, and has contributed to more balanced divisions of labor and resource use [19]. A similar lack of gender influence on the knowledge richness of useful medicinal plant was found in a Brazilian traditional community [15], as well as in a global scale [12]. In our study, the process by which people in the community acquired their plant knowledge may have contributed to gender similarity in plant uses. Women commonly told us that they learned about distinct plant groups while growing up, as they helped their father or mother in the field during subsistence or share-crop farming, or when collecting firewood for charcoal production. Two examples illustrate that both parents taught their children more or less equally to recognize useful plants:

*“We were little when we went with dad to the fields*. *We were already using the hoe*. *We also helped mom gather firewood*. *With all that*, *we grew up learning about plants*.*”* (interviewed woman)

*“My dad made charcoal to sell in Ouro Preto*. *And we had to help him*. *So*, *he taught us the kinds of woods that were good and bad for making charcoal*.*”* (interviewed woman)

The debate about the notions of labor, production, and reproduction in different cultural contexts that involve questions of gender roles and social anthropology is well known (e.g., [27]). The common identification of women as “homemakers” should be challenged, as women typically do much more than just run the home, including planting, harvesting, processing, and using woods for commercial exchanges or purchases, and other activities that do not fit under the “homemaker” umbrella [12,13,27], as evidenced by one of the interviewed woman:

*“I’m a seamstress*. *But I also gather and chop firewood*, *plant*, *and clean the pasture*. *I*, *with scythe in hand*, *am not envious of any man*, *swinging the scythe from side to side*, *like this*. *I also plow with a bull*. *I do just about anything*. *Where I used to live*, *whatever the men did*, *I did*, *alongside them and my dad*.*”* (interviewed woman)

In contradiction to most studies performed in patriarchal societies, including Brazil and Latin America in general, today, men and their wives in Brazilian urban communities tend to share all aspects of raising their children, along with other activities typically considered domestic and therefore associated with women [62]. Indeed, we recorded that many of the interviewed men are very knowledgeable about medicinal and edible plants, which suggests to us that in urban communities, despite the culture of historical patriarchal influence, there seems to be less of a gender division of labor with respect to plant resources. Here, passages from two interviewed men certainly illustrate the observed pattern:

*“We used plants for home remedies since I was little*. *My mom used to make all kinds of teas for us*. *Later*, *along come our own kids and grandkids*, *and like they say*, *we kept on using what we learned*.*"* (interviewed man)

“*If any kid has a stomach ache at home*, *I run to the garden and grab some ‘marcela’ (Achyrocline satureioides) for them to take*.*”* (interviewed man).

While we found that the genders have some similarity in known plants (i.e., inside each use category), important gender differences remained when we analyzed the distribution of TEK in the community, which illustrates differences in how this knowledge is shared. Plant repertories were more heterogeneous among men (greater β-diversity), suggesting to be due to the greater horizontal transmission of traditional knowledge about plants observed among women. Evidence from our observer-participant experiences suggests a high prevalence of horizontal transmission (teaching among neighbors and friends) of ethnobotanical information, where the person teaching exerts a very positive and long-lasting influence on the learner who then applies that knowledge in their daily life; a very important documented way of TEK conservation in traditional communities [63]. Indeed, during observer-participation in the studied community we noted much stronger associations among women, through both family ties and friendships. In fact, we observed that they often visit each other to exchange plants and information. Women also interact more frequently in other forms and purposes, such as religious or to make handicrafts, when they spend time also talking about other subjects. We also noted in our interviews that women that are more knowledgeable about plants in this community like to share this information. We did not observe this pattern on the interviewed men. One important implication of this closer relationship is that women teach each other more than men do. Thus, horizontal transfer of information is an important component of gender differences in the studied suburban community.

Even though both women and men say that vertical (parent-offspring) transmission of knowledge was the most important path of TEK acquisition, that importance may often be overestimated in survey interviews [9], such as in this study. It is suggested that the importance of horizontal and oblique transmission always increases when informants are not asked about who they learned from [64]. In addition, if a person learned about a species by vertical transmission, it can be later reinforced by horizontal sharing, influencing its use and conservation in the repertoire (e.g., the efficiency of a giving species to treat a disease). Here, a passage that illustrate the observed pattern:

"*I learned from my mother to use ‘macelinha’ since I was a child*. *Here in the community everyone has this plant at home*. *There is no better medicine for children’s diarrhea*.*"* (interviewed woman talking about *Chamaemelum nobile*)

The network comprising women and their known plants was more connected (greater connectance), with more knowledge sharing (lower modularity and higher niche overlap), and higher cohesion by central individuals that act as bridges, connecting distinct members in the community. Indeed, we found that the majority of woman (~94%) can be considered to be key-individuals (i.e., central in the network) in structuring plant knowledge of the entire community. Similar patterns were found in a Spanish traditional community by analyzing its landrace seed-exchange networks, with the women’s network having greater centrality than the men’s [59]. The female stereotype suggests that women are more extrovert and better able, or more inclined, to share sentiments, emotions and life experiences (including what they learn about plants) than men [25,65]. Sharing information among women is stimulated by a greater interaction among equals, as well as by a greater sensitivity to perceive biological phenomena, which is probably associated with motherhood [66]. Thus, we can infer that gender shapes the social-ecological network and the cultural sphere of the distribution and transmission of ethnobotanical knowledge.

While patterns of communication within and among genders varies among cultures [67], women in different communities worldwide, including urban Brazilian communities, tend towards a more structured social relationship among themselves [66]. The social structure we observed, i.e., a more cohesive network with more central individuals, suggests that these women share among themselves much more of their knowledge than do men. Horizontal transmission of knowledge and practices is elemental for the maintenance of TEK [8,10]. Typically, horizontal sharing of information mostly occurs among trusted friends and confidants, and those tend to be of the same gender [11,68,69]. Friendship among women is important for mutual quality and enjoyment of life [68], and so that which they learn for themselves or family care is also freely shared among others (see [63]). In fact, several interviews illustrated that women seem to exchange information, and other topics, more often than do men:

“*Eliana is my kid’s godmother and we’re both curious about plants and their uses*, *and so when one of us finds a new plant*, *we tell the other right away*.” (interviewed woman)

“*I wasn’t familiar with this plant*, *and my neighbor gave me a few leaves to make some tea when my little boy was sick*. *It was great*! *So*, *I started growing it and now we’re never without it*.” (interviewed woman talking about *Sambucus australis*)

Traditions of plant uses sharing among community members depend on the nature of information that is learned and shared informally and socially [63]. Here, we provide additional emphasis of the importance of gender in shaping the structure of social-ecological networks and the importance of woman in connecting members and sharing traditional knowledge of plants in the community. It is important to remember that our study comprises a sample of a suburban tropical community, with most members presenting low incomes and limited formal education. Science has an interest in identifying patterns of people and natural resources relationship on a global scale [70]. We expect that the patterns and behaviors of our study would be highly similar in other cultures and regions. However, similar approaches in different environmental and social-cultural conditions, as well as a macro-ethnoecological approach are necessary for a better understanding of gender patterns that are tied to the use of natural resources and knowledge transmission [70]. Our study brings some important findings about how the art of communication and how information exchange prompts distinct network structures with different patterns of knowledge diversity.

Social ecological system resilience has been a new concern on ethnobiology [11,71,72]. Transmission of knowledge has been stated as able to model TEK diversity [13,72], influencing the resilience of the traditional knowledge [11,13]. The resilience of social-ecological system would be reduced if the system is restricted to one or few persons [72], i.e., when the community presents a higher beta-diversity. In addition, the higher network connectivity due to the higher TEK transmission is related with the functionality of social-ecological systems, contributing to their resilience [13]. Thus, the proposal of network cohesiveness and diversity metrics of our study seems to be a good tool for a better understanding of TEK diversity into gender groups in order to contribute to the comprehension of social ecological-system resilience. In this sense, in a community with more homogeneous TEK, if a member is out of the system another one can play a similar function, which would contribute to TEK conservation. In our case, women proved to be very important to guarantee this system resilience.

Our study also raises some new questions about network cohesiveness and TEK diversity. For example, in a broader scale is there a greater cohesiveness of woman ethnobotanical network? Which factors able to model gender behavior (e.g., biological, psychological, cultural, economic, and environmental) interfere in knowledge transmission? To answer these and other questions that encompass the division of knowledge and its transmission, continued research is required in a variety of socio-cultural conditions. These questions would lead us to a better understanding of the social-ecological systems. Understanding that gender plays such an important role in the perception and transmission of TEK can guide future studies as well as governance strategies for conservation of this important knowledge and plant resources.

## Supporting information

S1 Table Species and families of useful plants and their popular names, categories of use, voucher number and code number identified in an ethnobotanical survey in a suburban community of Ouro Preto, Minas Gerais, Brazil. (DOCX)

S2 Table Comparison of connectance (C), niche overlap in resource use (Horn), modularity (Q), and their respective significances (N = 999 randomizations) for woman and man ethnobotanical networks from a suburban community, Ouro Preto, Brazil. *Indicate metrics significance against Patefield null model; St. = Standardized values for each metric considering the number of standard deviations above the average value recorded in 999 randomizations. Standardized values lower or greater than two indicate significant values, since they represent how many standard deviations the real observed metric is far from the mean of 999 values generated from randomized networks. Therefore, instead of P-values, we used standardized values to estimate metrics’ significance.
(DOCX)

## References

[1] Voeks RA. Are women reservoirs of traditional plant knowledge? Gender, ethnobotany and globalization in northeast Brazil. Singap J Trop Geogr. 2007;28: 7–20. doi: 10.1111/j.1467-9493.2006.00273.x

[2] Reyes-García V, Broesch J, Calvet-Mir L, Fuentes-Peláez N, McDade TW, Parsa S, et al. Cultural transmission of ethnobotanical knowledge and skills: an empirical analysis from an Amerindian society. Evol Hum Behav. 2009;30: 274–285. doi: 10.1016/j.evolhumbehav.2009.02.001

[3] Albuquerque UP, Medeiros PM, Ferreira Júnior WS, Silva TC, Silva RRV, Gonçalves-Souza T. Social-Ecological Theory of Maximization: Basic concepts and two initial models. Biol Theory. 2019;14: 73–85. doi: 10.1007/s13752-019-00316-8

[4] Albuquerque UP, Nascimento ALB, Soldati GT, Feitosa IS, Campos JLA, Hurrell JA, et al. Ten important questions/issues for ethnobotanical research. Acta Bot Bras. 2019;33: 376–385. doi: 10.1590/0102-33062018abb0331

[5] Mesoudi A, Whiten A, Laland KN. Towards a unified science of cultural evolution. Behav Brain Sci. 2006;29: 329–347. doi: 10.1017/S0140525X06009083 17094820

[6] Soldati GT, Hanazaki N, Crivos M, Albuquerque UP. Does environmental instability favor the production and horizontal transmission of knowledge regarding medicinal plants? A study in southeast Brazil. Flynn E, editor. PLoS One. 2015;10: e0126389. doi: 10.1371/journal.pone.0126389 25992578 PMC4439025

[7] Boyd R, Richerson PJ, Henrich J. The cultural niche: Why social learning is essential for human adaptation. Proc Natl Acad Sci. 2011;108: 10918–10925. doi: 10.1073/pnas.1100290108 21690340 PMC3131818

[8] Lozada M, Ladio A, Weigandt M. Cultural transmission of ethnobotanical knowledge in a rural community of Northwestern Patagonia, Argentina. Econ Bot. 2006;60: 374–385. doi: 10.1663/0013-0001(2006)60[374:CTOEKI]2.0.CO;2

[9] Soldati GT, Albuquerque UP. Are the evolutionary implications of vertical transmission of knowledge conservative? Ethnobiol Conserv. 2016;5: 1–9. doi: 10.15451/ec2016-6-5.2-1-09

[10] Eyssartier C, Ladio AH, Lozada M. Cultural transmission of traditional knowledge in two populations of North-western Patagonia. J Ethnobiol Ethnomed. 2008;4: 25. doi: 10.1186/1746-4269-4-25 19077315 PMC2614966

[11] Torres-Avilez W, Nascimento ALB, Santoro FR, Medeiros PM, Albuquerque UP. Gender and its role in the resilience of local medical systems of the Fulni-ô people in NE Brazil: Effects on structure and functionality. Evidence-based Complement Altern Med. 2019;2019. doi: 10.1155/2019/8313790 31281403 PMC6594245

[12] Torres-Avilez W, Medeiros PM, Albuquerque UP. Effect of gender on the knowledge of medicinal plants: Systematic review and meta-analysis. Evidence-Based Complement Altern Med. 2016;2016: 1–13. doi: 10.1155/2016/6592363 27795730 PMC5067321

[13] Torres-Avilez WM, Albuquerque UP. Dynamics of socialecological systems: Gender influence in local medical systems. Ethnobiol Conserv. 2017;6: 1–6. doi: 10.15451/ec2017-076.8-1-6

[14] Müller JG, Boubacar R, Guimbo ID. The “How” and “Why” of including gender and age in ethnobotanical research and community-based resource management. Ambio. 2015;44: 67–78. doi: 10.1007/s13280-014-0517-8 24789508 PMC4293356

[15] Alencar NL, Júnior WSF, Albuquerque UP. Medicinal plant knowledge richness and sharing in Northeastern Brazil. Econ Bot. 2014;68: 371–382. doi: 10.1007/s12231-014-9284-5

[16] Albuquerque UP, Ramos MA, Lucena RFP, Alencar NL. Methods and techniques used to collect ethnobiological data. Methods and techniques in ethnobiology and ethnoecology. New York, USA: Springer; 2014. pp. 15–38. doi: 10.1007/978-1-4614-8636-7

[17] Pfeiffer JM, Butz RJ. Assessing cultural and ecological variation in ethnobiological research: The importance of gender. J Ethnobiol. 2005;25: 240–278. doi: 10.2993/0278-0771(2005)25[240:ACAEVI]2.0.CO;2

[18] Di Ciommo RC. Pescadoras e pescadores: a questão da equidade de gênero em uma reserva extrativista marinha. Ambient Soc. 2007;10: 151–163. doi: 10.1590/S1414-753X2007000100010

[19] Ladio AH. La etnobiología en áreas rurales y su aporte a la lucha para desentrañar sesgos patriarcales. Ethnoscientia. 2020;5: 1–13. doi: 10.22276/ethnoscientia.v5i1.298

[20] Kainer KA, Duryea ML. Tapping women’s knowledge: Plant resource use in extractive reserves, Acre, Brazil. Econ Bot. 1992;46: 408–425. doi: 10.1007/BF02866513

[21] Camou-Guerrero A, Reyes-García V, Martínez-Ramos M, Casas A. Knowledge and use value of plant species in a Rarámuri community: A gender perspective for conservation. Hum Ecol. 2008;36: 259–272. doi: 10.1007/s10745-007-9152-3

[22] Reyes-García V, Vila S, Aceituno-Mata L, Calvet-Mir L, Garnatje T, Jesch A, et al. Gendered homegardens: A study in three mountain areas of the Iberian Peninsula. Econ Bot. 2010;64: 235–247. doi: 10.1007/s12231-010-9124-1

[23] Dovie DBK, Witkowski ETF, Shackleton CM. Knowledge of plant resource use based on location, gender and generation. Appl Geogr. 2008;28: 311–322. doi: 10.1016/j.apgeog.2008.07.002

[24] Howard P. Women and the plant world: An exploration. In: Howard PL, editor. Women & Plants: Gender relations in biodiversity management & conservation. London & New York: Zed Press and Palgrave-Macmillan; 2003. pp. 4–34.

[25] Wood JT, Inman CC. In a different mode: Masculine styles of communicating closeness. J Appl Commun Res. 1993;21: 279–295. doi: 10.1080/00909889309365372

[26] Kéfi S, Berlow EL, Wieters EA, Navarrete SA, Petchey OL, Wood AB, et al. More than a meal… integrating non-feeding interactions into food webs. Ecol Lett. 2012;15: 291–300. doi: 10.1111/j.1461-0248.2011.01732.x 22313549

[27] Moore HL. Antropología y Feminismo. 5th ed. Madrid ES: Ediciones Cátedra; 2004.

[28] Elias M. Gender, knowledge-sharing and management of shea (*Vitellaria paradoxa*) parklands in central-west Burkina Faso. J Rural Stud. 2015;38: 27–38. doi: 10.1016/j.jrurstud.2015.01.006

[29] Magurran AE. Measuring biological diversity. Oxford, United Kingdom: Wiley-Blackwell; 2004.

[30] Lopes VL, Costa FV, Rodrigues RA, Braga EM, Pichorim M, Moreira PA. High fidelity defines the temporal consistency of host-parasite interactions in a tropical coastal ecosystem. Sci Rep. 2020;10: 16839. doi: 10.1038/s41598-020-73563-6 33033317 PMC7545182

[31] Costa FV, Mello MAR, Bronstein JL, Guerra TJ, Muylaert RL, Leite AC, et al. Few ant species play a central role linking different plant resources in a network in rupestrian grasslands. Nascimento FS, editor. PLoS One. 2016;11: e0167161. doi: 10.1371/journal.pone.0167161 27911919 PMC5135051

[32] Mello MAR, Rodrigues FA, Costa LF, Kissling WD, Şekercioğlu ÇH, Marquitti FMD, et al. Keystone species in seed dispersal networks are mainly determined by dietary specialization. Oikos. 2015;124: 1031–1039. doi: 10.1111/oik.01613

[33] Begossi A, Hanazaki N, Tamashiro JY. Medicinal plants in the Atlantic Forest (Brazil): Knowledge, use, and conservation. Hum Ecol. 2002;30: 281–299. doi: 10.1023/A:1016564217719

[34] UNESCO. World heritage convention: Historic town of Ouro Preto. 2020 [cited 20 Oct 2020]. Available: https://whc.unesco.org/en/list/124.

[35] Messias MCTB, Menegatto MF, Prado ACC, Santos BR, Guimarães MFM. Uso popular de plantas medicinais e perfil socioeconômico dos usuários: um estudo em área urbana em Ouro Preto, MG, Brasil. Rev Bras Plantas Med. 2015;17: 76–104. doi: 10.1590/1983-084X/12_139

[36] IBGE. Instituto Brasileiro de Geografia e Estatística. Cities and State. [cited 20 Oct 2020]. Available: https://www.ibge.gov.br/en/cities-and-states.html.

[37] Rizzini CT. Tratado de fitogeografia do Brasil. 2nd ed. Rio de Janeiro: Âmbito Cultural; 1997.

[38] Alvares CA, Stape JL, Sentelhas PC, Gonçalves JLM, Sparovek G. Köppen’s climate classification map for Brazil. Meteorol Zeitschrift. 2013;22: 711–728. doi: 10.1127/0941-2948/2013/0507

[39] Urriago-Ospina LM, Jardim CM, Rivera-Fernández G, Kozovits AR, Leite MGP, Messias MCTB. Traditional ecological knowledge in a ferruginous ecosystem management: lessons for diversifying land use. Environ Dev Sustain. 2021;23: 2092–2121. doi: 10.1007/s10668-020-00665-6

[40] Tongco MDC. Purposive sampling as a tool for informant selection. Ethnobotany research and applications. Ethnobot Res Appl. 2007;5: 147–158. Available: www.ethnobotanyjournal.org/vol5/i1547-3465-05-147.pdf.

[41] Albuquerque UP, Lucena RFP, Lins Neto EMF. Selection of research participants. Methods and techniques in ethnobiology and ethnoecology. New York, USA: Springer; 2014. pp. 1–14. doi: 10.1007/978-1-4614-8636-7

[42] Bailey K. Methods of social research. New York, USA: The Free Press; 1994.

[43] Peixoto AL, Maia LC. Manual de procedimentos para herbários. Recife, Brazil: Editora Universitária UFPE; 2013.

[44] Chase MW, Reveal JL. A phylogenetic classification of the land plants to accompany APG III. Bot J Linn Soc. 2009;161: 122–127. doi: 10.1111/j.1095-8339.2009.01002.x

[45] The Plant List. The plant list: A working list of all plant species. 2013.

[46] Zar JH. Biostatistical Analysis. 4th ed. Prentice-Hall, New Jersey; 1999.

[47] Whittaker R. Vegetation of the Siskiyou Mountains, Oregon and California. Ecol Monogr. 1960;30: 279–338.

[48] Dormann CF, Strauss R. A method for detecting modules in quantitative bipartite networks. Peres-Neto P, editor. Methods Ecol Evol. 2014;5: 90–98. doi: 10.1111/2041-210X.12139

[49] Manly BFJ. Randomization, Bootstrap and Monte Carlo Methods in Biology. 3rd ed. Chapman and Hall; 2007.

[50] Dormann CF, Fruend J, Gruber B, Devoto M, Iriondo J, Strauss R, et al. Visualising bipartite networks and calculating some (ecological) indices. Vegan, sna. 2014; 149.

[51] R Core Team. R: A language and environment for statistical computing. Viena, Austria: R Foundation for Statistical Computing; 2020. 3-900051-07-0.

[52] Batagelj V, Mrvar A. Pajek–a program for large network analysis. Connections. 1998;21: 47–57. Available: http://vlado.fmf.uni-lj.si/pub/networks/pajek/.

[53] Stehmann JR, Brandão MGL. Medicinal plants of Lavras Novas (Minas Gerais, Brazil). Fitoterapia. 1995;66: 515–520.

[54] Prado ACC, Rangel EB, Sousa HC, Messias MCTB. Etnobotânica como subsídio à gestão socioambiental de uma unidade de conservação de uso sustentável. Rodriguésia. 2019;70: e02032017. doi: 10.1590/2175-7860201970019

[55] Url S, Rossato SC. Ethnobotany of Caiçaras of the Atlantic Forest coast (Brazil). Econ Bot. 2013;53: 387–395.

[56] Hanazaki N, Tamashiro JY, Leitão-Filho HF, Begossi A. Diversity of plant use in two Caiçara communities from the Atlantic Forest coast, Brazil. Biodivers Conserv. 2000;9: 597–615.

[57] Barbosa KA, Souza LF, Silva FG, Vitorino LC, Bessa LA, Menino GCO, et al. Quilombola ethnobotany: A case study in a community of slave descendants from the center of the Cerrado biome. Res Soc Dev. 2020;9: e332985797. 10.33448/rsd-v9i8.5797 Maria.

[58] Albuquerque UP, Holanda C. Andrade L. Ethnobotany of the genus *Ocimum* L. (*Lamiaceae*) by Afrobrasilian communities. An del Jardín Botánico Madrid. 1998;56. doi: 10.3989/ajbm.1998.v56.i1.224

[59] Calvet-Mir L, Calvet-Mir M, Molina JL, Reyes-García V. Seed Exchange as an agrobiodiversity conservation mechanism. A Case Study in Vall Fosca, Catalan Pyrenees, Iberian Peninsula. Ecol Soc. 2012;17: art29. doi: 10.5751/ES-04682-170129

[60] Heineberg MR, Hanazaki N. Dynamics of the botanical knowledge of the Laklãnõ-Xokleng indigenous people in Southern Brazil. Acta Bot Brasilica. 2019;33: 254–268. doi: 10.1590/0102-33062018abb0307

[61] Luoga EJ, Witkowski ETE, Balkwill K. Differential utilization and ethnobotany of trees in Kitulanghalo Forest Reserve and surrounding communal lands, Eastern Tanzania. Econ Bot. 2000;54: 328–343.

[62] Schaedel RP, Hardoy JE, Scott-Kinzer N. Urbanization in the Americas from its beginning to the present. Chicago, IL, USA: Aldine Publishing Company; 2012. doi: 10.1515/9783110808018

[63] Hoppitt W, Laland KN. Social Learning: An Introduction to Mechanisms, Methods, and Models. Oxford, United Kingdom: Princeton UP; 2013.

[64] Henrich J, Broesch J. On the nature of cultural transmission networks: evidence from Fijian villages for adaptive learning biases. Philos Trans R Soc B Biol Sci. 2011;366: 1139–1148. doi: 10.1098/rstb.2010.0323 21357236 PMC3049092

[65] Onnela J-P, Waber BN, Pentland A, Schnorf S, Lazer D. Using sociometers to quantify social interaction patterns. Sci Rep. 2014;4: 5604. doi: 10.1038/srep05604

[66] Di Ciommo RC. Relações de gênero, meio ambiente e a teoria da complexidade. Rev Estud Fem. 2003;11: 423–443. doi: 10.1590/S0104-026X2003000200005

[67] Wood JT. Gendered Lives: Communication, gender, and culture. 9th ed. Boston: Wadsworth Cenage Learning; 2013.

[68] Sapadin LA. Friendship and gender: Perspectives of professional men and women. J Soc Pers Relat. 1988;5: 387–403.

[69] Samter W, Burleson BR. The role of communication in same-sex friendships: A comparison among African Americans, Asian Americans, and European Americans. Commun Q. 2005;53: 265–283. doi: 10.1080/01463370500100982

[70] Albuquerque UP, Medeiros PM. Systematic reviews and meta-analysis applied to ethnobiological research. Ethnobiol Conserv. 2012;1: 1–8.

[71] Berkes F, Folke C. Linking Social and Ecological Systems: Management Practices and Social Mechanisms for Building. Cambridge, UK: Cambridge Univ. Press.; 2000. doi: 10.1046/j.1524-4725.2000.00021.x

[72] Ferreira-Júnior WS, Nascimento ALB, Ramos MA, Medeiros PM, Soldati GT, Santoro FR, et al. Resilience and adaptation in social-ecological systems. In: Albuquerque UP, Medeiros PM, Casas A, editors. Evolutionary Ethnobiology. New York: Springer International Publishing; 2015. pp. 105–119. doi: 10.1007/978-3-319-19917-7_8

