# Supplementary material for: Gender differences in traditional knowledge of useful plants in a Brazilian community
Source: PLoS One. 2021 Jul 28;16(7):e0253820. doi: 10.1371/journal.pone.0253820 (PMC8318302; doi:10.1371/journal.pone.0253820)
Supplement: S1 Table — (DOCX) [file pone.0253820.s001.docx]

S1 Table. List of plant species and their respective families, Brazilian popular name, use category, voucher (OUPR Herbarium accession number) and code numbers (cited in networks’ drawings), from an ethnobotanical study, in the municipality of Ouro Preto, Minas Gerais, Brazil. NC: not collected.

| **Family**/Species | **Popular name** | **Use** | **Voucher** | **Code** |
| --- | --- | --- | --- | --- |
| **Acanthaceae** |  |  |  |  |
| *Aphelandra colorata* (Vell.) Wassh. | Suspiro | Ornamental | 2710 | 1 |
| *Pachystachys lutea* Nees | Camarão | Ornamental | 2711 | 2 |
| **Agavaceae** |  |  |  |  |
| *Furcraea foetida* (L.) Haw. | Piteira | Medicinal/ Technological | 2712 | 3 |
| **Alismataceae** |  |  |  |  |
| *Echinodorus macrophyllus* (Kunth) Micheli | Chapeu-de-couro | Medicinal | 2713 | 4 |
| **Amaranthaceae** |  |  |  |  |
| *Amaranthus viridis* L. | Caruru-de-porco | Edible /Medicinal | 28848 | 5 |
| *Amaranthus* sp. | Carurú | Edible /Medicinal | NC | 6 |
| *Dysphania ambrosioides* (L.) Mosyakin & Clemants | Santa-maria | Medicinal | 28887 | 7 |
| *Gomphrena globosa* L. | Perpétua | Medicinal | 29152 | 8 |
| *Iresine herbstii* Hook. | Cruzeira-vermelha | Medicinal | 28900 | 9 |
| *Pfaffia glomerata (*Spreng.) Pedersen | Dipirona | Medicinal | 29151 | 10 |
| **Amaryllidaceae** |  |  |  |  |
| *Allium cepa* L. | Cebola | Edible/Medicinal | NC | 11 |
| *Allium sativum* L. | Alho | Edible/Medicinal | NC | 12 |
| *Allium fistulosum* L. | Cebolinha | Edible/Medicinal | NC | 13 |
| *Hippeastrum reginae* (L.) Herb. | Lírio-vermelho | Ornamental | 2714 | 14 |
| **Anacardiaceae** |  |  |  |  |
| *Mangifera indica* L. | Manga | Edible | NC | 15 |
| *Myracrodruon urundeuva* Allemão | Aroeira-do-sertão | Fuel/Medicinal | 29153 | 16 |
| *Schinus terebinthifolia* Raddi | Aroeira | Edible/ Fuel/Ecological/Timber/Medicinal/ Technological | 28961 | 17 |
| **Annonaceae** |  |  |  |  |
| *Annona muricata* L. | Nona | Edible/ Medicinal | 29154 | 18 |
| *Rollinia sylvatica (A.St.-Hil.) Mart* | Araticum | Edible | 2739 | 19 |
| *Xylopia brasiliensis* Spreng. | Pindaíba | Fuel/Timber | 29155 | 20 |
| *Xylopia sericea* A.St.-Hil. | Embira | Fuel/Timber/ Technological | 29156 | 21 |
| **Apiaceae** |  |  |  |  |
| *Apium graveolens* L. | Aipo | Edible/Medicinal | NC | 22 |
| *Centella asiatica* (L.) Urb. | Capitão | Medicinal | 28873 | 23 |
| *Daucus carota* L. | Cenoura | Edible/Medicinal | NC | 24 |
| *Ferula assa-foetida* L. | Assa-fétida | Medicinal | 28985 | 25 |
| *Foeniculum vulgare* Mill. | Funcho | Medicinal | 28891 | 26 |
| *Petroselinum crispum* (Mill.) Fuss | Salsa | Edible/Cosmetic/Medicinal | 29157 | 27 |
| **Apocynaceae** |  |  |  |  |
| *Aspidosperma* sp. | Peroba | Fuel/Timber/Medicinal/ Technological | 29158 | 28 |
| **Aquifoliaceae** |  |  |  |  |
| *Ilex chamaedryfolia*Reissek | Congonha-folha-miúda | Medicinal | 28984 | 29 |
| **Araceae** |  |  |  |  |
| *Anthurium x froebelii* Hort. | Antúrio | Ornamental | 2715 | 30 |
| *Colocasia esculenta* (L.) Schott | Inhame | Edible/Medicinal | 29159 | 31 |
| *Dieffenbachia seguine* (Jacq.) Schott | Comigo-ninguém-pode | Mystical/Ornamental | 29160 | 32 |
| *Philodendron* sp. | Imbé | Ornamental | 29161 | 33 |
| *Xanthosoma taioba* E.G.Gonç. | Taioba | Edible/Medicinal | 29162 | 34 |
| **Arecaceae** |  |  |  |  |
| *Euterpe oleracea* Mart. | Açaí | Edible/ Technological | NC | 35 |
| **Asparagaceae** |  |  |  |  |
| *Sansevieria trifasciata* Prain | Espada-de-são-jorge | Mystical/Ornamental | 2716 | 36 |
| **Asteraceae** |  |  |  |  |
| *Acanthospermum australe* (Loefl.) Kuntze | Carrapichinho | Medicinal | 29164 | 37 |
| *Achillea millefolium* L. | Mil-folhas | Medicinal | 28839 | 38 |
| *Achyrocline satureioides* (Lam.) DC. | Marcela | Medicinal/ Ornamental/ Technological | 28840 | 39 |
| *Ageratum conyzoides* (L.) L. | São-joão | Medicinal | 28844 | 40 |
| *Arctium lappa* L. | Bardana | Edible/Medicinal | 28853 | 41 |
| *Artemisia absinthium* L. | Losna | Medicinal | 28854 | 42 |
| *Artemisia alba* Turra | Alcanfôr | Medicinal | 28855 | 43 |
| *Austroeupatorium inulaefolium* (Kunth) R.M.King & H.Rob. | Porrete | Medicinal | 28857 | 44 |
| *Baccharis crispa* Spreng. | Carqueja | Cosmetic/Medicinal | 28859 | 45 |
| *Baccharis dracunculifolia* DC. | Alecrim-do-campo | Fuel/Medicinal/Mystical / Technological | 28861 | 46 |
| *Bidens pilosa* L. | Picão | Edible/Medicinal/Mystical | 28864 | 47 |
| *Chamaemelum nobile* (L.) All. | Macelinha | Medicinal | 29165 | 48 |
| *Chaptalia nutans* (L.) Polák | Lanceta | Medicinal | 28875 | 49 |
| *Cosmos caudatus* Kunth | Avoadeira | Medicinal | 2733 | 50 |
| *Cynara scolymus* L. | Alcachofra | Edible/Medicinal | 29167 | 51 |
| *Dendranthema grandiflorum* (Ramat.) Kitam. | Monsenhor | Ornamental | 28885 | 52 |
| *Erechtites hieraciifolius* (L.) Raf. ex DC. | Maria-arnica. carurú-arnica | Edible | 28889 | 53 |
| *Eremanthus erythropappus* (DC.) MacLeish | Candeia | Fuel/ Cosmetic/Timber/Medicinal | 29168 | 54 |
| *Eremanthus glomeratus* Less. | Candeião | Fuel | 29169 | 55 |
| *Gymnanthemum amygdalinum* (Delile) Sch.Bip. ex Walp. | Boldo-do-sertão | Medicinal | 28896 | 56 |
| *Lactuca sativa* L. | Alface | Edible/Medicinal | NC | 57 |
| *Lychnophora pinaster* L. | Arnica | Medicinal | 29170 | 58 |
| *Mikania glomerata* Spreng. | Guaco | Medicinal | 29172 | 59 |
| *Mikania hirsutissima* DC. | Cipó-cabeludo | Medicinal | 28920 | 60 |
| *Smallanthus sonchifolius* (Poepp.) H.Rob. | Batata-iacom | Edible/Medicinal | 28967 | 61 |
| *Sonchus oleraceus* (L.) L. | Serralha | Edible | 29174 | 62 |
| *Symphyopappus decemflorus* H.Rob. | Pustemão | Medicinal | 28982 | 63 |
| *Symphyotrichum squamatum* (Spreng.) G.L.Nesom | Quebra-junta | Medicinal | 28983 | 64 |
| *Synedrella nodiflora* (L.) Gaertn. | Canela-de-urubú | Medicinal | 29175 | 65 |
| *Tagetes erecta* L. | Cravo-de-defunto | Medicinal | 28986 | 66 |
| *Tanacetum vulgare* L. | Pluma | Medicinal | 28987 | 67 |
| *Taraxacum campylodes* G.E.Haglund | Dente-de-leão | Edible/Medicinal | 28989 | 68 |
| *Trixis antimenorrhoea* (Schrank) Mart. ex Baker | Solidônia | Medicinal | 2719 | 69 |
| *Vernonanthura phosphorica* (Vell.) H.Rob. | Assapeixe | Edible/Fuel/Medicinal | 28993 | 70 |
| **Begoniaceae** |  |  |  |  |
| *Begonia angularis* Raddi | Sete-sangrias | Medicinal | 28863 | 71 |
| *Begonia ulmifolia* Willd. | Begônia | Ornamental | 29177 | 72 |
| **Bignoniaceae** |  |  |  |  |
| *Handroanthus chrysotrichus* (Mart. ex DC.) Mattos | Ipê-amarelo | Technological/Medicinal | 2720 | 73 |
| *Jacaranda caroba* (Vell.) DC. | Carobinha | Medicinal | 28901 | 74 |
| *Podranea ricasoliana* (Tanfani) Sprague | Sete-leguas | Ornamental | 28945 | 75 |
| *Pyrostegia venusta* (Ker Gawl.) Miers | Cipó-de-são-joão | Medicinal/ Technological | 28950 | 76 |
| *Tynanthus elegans* Miers | Cipó-cravo | Technological | 2720 | 77 |
| **Bixaceae** |  |  |  |  |
| *Bixa orellana* L. | Urucum | Edible/ Technological/Medicinal | 28865 | 78 |
| **Boraginaceae** |  |  |  |  |
| *Symphytum officinale* L. | Confrei | Medicinal | 29180 | 79 |
| **Brassicaceae** |  |  |  |  |
| *Brassica oleracea* L. | Couve | Edible/Medicinal | 28866 | 80 |
| *Brassica rapa* L. | Nabo | Edible/Medicinal | NC | 81 |
| *Lepidium didymum* L. | Mentruz | Edible | 28909 | 82 |
| *Nasturtium officinale* R.Br. | Agrião | Edible/Medicinal | 29181 | 83 |
| *Raphanus raphanistrum* subsp. *sativus* (L.) Domin | Rabanete | Edible | NC | 84 |
| **Cactaceae** |  |  |  |  |
| *Pereskia aculeata* Mill. | Ora-pro-nobis, lobrobrô | Edible/Medicinal/Mystical | 29182 | 85 |
| **Caprifoliaceae** |  |  |  |  |
| *Sambucus australis* Cham. & Schltdl. | Sabugueiro | Medicinal | 29183 | 86 |
| **Caricaceae** |  |  |  |  |
| *Carica papaya* L. | Mamão | Edible/Medicinal/ Technological | NC | 87 |
| **Caryophyllaceae** |  |  |  |  |
| *Dianthus caryophyllus* L. | Cravina | Mystical/Ornamental | 29184 | 88 |
| **Clusiaceae** |  |  |  |  |
| *Garcinia brasiliensis* Mart. | Bacupari | Edible | 29185 | 89 |
| *Vismia brasiliensis* Choisy | Casca-de-barata | Fuel | 29186 | 90 |
| **Commelinaceae** |  |  |  |  |
| *Callisia repens* (Jacq.) L. | Dinheiro-em-penca | Mystical | 29187 | 91 |
| *Tripogandra serrulata* (Vahl) Handlos | Trapoeraba | Fodder | 29188 | 92 |
| **Convolvulaceae** |  |  |  |  |
| *Ipomoea batatas* (L.) Lam. | Batata-doce | Edible/Medicinal | 29190 | 93 |
| *Ipomoea cairica* (L.) Sweet | Cipó-cinco-folhas | Medicinal/ Technological | 29191 | 94 |
| *Ipomoea purpurea* (L.) Roth. | Cipó-batata | Medicinal/ Technological | 29189 | 95 |
| **Costaceae** |  |  |  |  |
| *Costus comosus* (Jacq.) Roscoe | Pacová | Medicinal | 2723 | 96 |
| *Costus spicatus* (Jacq.) Sw. | Cana-de-macaco | Medicinal | 29192 | 97 |
| *Costus spiralis* (Jacq.) Roscoe | Cana-de-macaco | Medicinal | 28880 | 98 |
| **Crassulaceae** |  |  |  |  |
| *Bryophyllum pinnatum* (Lam.) Oken | Flor-da fortuna | Medicinal/Ornamental | 29193 | 99 |
| *Echeveria elegans* Rose | Rosa-de-pedra | Ornamental | 2724 | 100 |
| *Kalanchoe laciniata* (L.) DC. | Saião | Medicinal/Ornamental | 28902 | 101 |
| *Sedum dendroideum* Moc. & Sessé ex DC. | Basto, baspo | Medicinal | 28964 | 102 |
| **Cucurbitaceae** |  |  |  |  |
| *Cucumis sativus* L. | Pepino | Medicinal/Edible/ Medicinal | NC | 103 |
| *Cucurbit*a sp. | Abóbora | Edible | NC | 104 |
| *Cyclanthera pedata* (L.) Schrad. | Maxixe-liso | Edible | 28882 | 105 |
| *Melothrianthus smilacifolius* (Cogn.) Mart.Crov. | Catingueira, cipó-azougue | Medicinal | 28852 | 106 |
| *Momordica charantia* L. | Melão-de-são-caetano | Medicinal | 28921 | 107 |
| *Sechium edule* (Jacq.) Sw. | Chuchu | Edible/Medicinal | 28963 | 108 |
| **Dennstaedtiaceae** |  |  |  |  |
| *Pteridium arachnoideum* (Kaulf.) Maxon | Samambaia-de-broto | Edible | 29197 | 109 |
| **Dicksoniaceae** |  |  |  |  |
| *Dicksonia sellowiana* Hook. | Xaxim | Edible/ Technological | 29198 | 110 |
| **Dilleniaceae** |  |  |  |  |
| *Davilla rugosa* Poir. | Cipó-carijó | Medicinal/ Technological | 28884 | 111 |
| **Dioscoreaceae** |  |  |  |  |
| *Dioscorea bulbifera* L. | Cará | Edible/Medicinal | 29200 | 112 |
| **Ebenaceae** |  |  |  |  |
| *Diospyros kaki* L.f. | Caqui | Edible | NC | 113 |
| **Equisetaceae** |  |  |  |  |
| *Equisetum arvense* L. | Cavalinha | Medicinal | 29201 | 114 |
| **Ericaceae** |  |  |  |  |
| *Rhododendron indicum* (L.) Sweet | Azaléia | Ornamental | 29202 | 115 |
| **Euphorbiaceae** |  |  |  |  |
| *Croton floribundus* Spreng. | Pixinguí | Fuel/Medicinal | 29203 | 116 |
| *Croton* sp. | Sangria-d´água | Fuel/Timber/Medicinal | 29204 | 117 |
| *Mabea fistulifera* Mart. | Canudo-de-pito | Fuel/Timber/Ecological | 29205 | 118 |
| *Manihot esculenta* Crantz | Mandioca | Edible/Medicinal | 28915 | 119 |
| *Ricinus communis* L. | Mamona | Fuel/Cosmetic/Medicinal | 29206 | 120 |
| *Sapium glandulosum* (L.) Morong | Pau-de-leite | Fuel | 29207 | 121 |
| **Fabaceae** |  |  |  |  |
| *Amburana cearensis* (Allemão) A.C.Sm. | Cumarú | Timber | NC | 122 |
| *Andira* sp. | Angelim | Fuel/Timber | 29208 | 123 |
| *Apuleia leiocarpa* (Vogel) J.F.Macbr. | Garapa | Fuel | 29209 | 124 |
| *Bauhinia cheilantha* (Bong.) Steud. | Pata-de-vaca | Medicinal | 29210 | 125 |
| *Bowdichia virgilioides* Kunth | Sucupira | Timber/Medicinal | 29211 | 126 |
| *Cajanus cajan* (L.) Millsp. | Feijão-andú | Edible/Ecological | 29212 | 127 |
| *Copaifera langsdorffii* Desf. | Copaíba, pau-d´óleo | Medicinal | 29213 | 128 |
| *Dalbergia nigra* (Vell.) Allemão ex Benth. | Jacarandá-cabiúna | Timber | 28883 | 129 |
| *Desmodium incanum* DC. | Carrapichinho | Medicinal/Forrageiro | 28886 | 130 |
| *Hymenaea* sp. | Jatobá | Medicinal | 29215 | 131 |
| *Indigofera suffruticosa* Mill. | Anileira | Technological/Ecological | 29216 | 132 |
| *Inga* sp. | Angá | Edible/Ecological/Medicinal | 28899 | 133 |
| *Machaerium* sp. | Pau-de-espinho | Fuel/Timber/Technological | 29217 | 134 |
| *Melanoxylon brauna* Schott | Braúna | Timber | NC | 135 |
| *Phaseolus lunatus* L. | Fava | Edible | 29214 | 136 |
| *Piptadenia gonoacantha* (Mart.) J.F.Macbr | Angico | Fuel/Medicinal/Timber | 29218 | 137 |
| *Plathymenia reticulata* Benth. | Vinhático | Technological/Timber | 29219 | 138 |
| *Platymiscium floribundum* Vogel | Jacarandá-vermelho | Timber | 29220 | 139 |
| *Senegalia polyphylla* (DC.) Britton & Rose | Monjolo | Fuel | 29221 | 140 |
| *Senna* sp. | Fedegoso | Medicinal | 29222 | 141 |
| *Stryphnodendron polyphyllum* Mart. | Barbatimão | Medicinal/ Technological | 28981 | 142 |
| *Vigna* sp. | Feijão-de-corda | Edible | 29223 | 143 |
| **Fagaceae** |  |  |  |  |
| *Castanea sativa* Mill. | Castanha | Edible | NC | 144 |
| *Quercus robur* L. | Carvalho | Timber | NC | 145 |
| **Geraniaceae** |  |  |  |  |
| *Pelargonium graveolens* (L.) L'Hér | Jardineira | Medicinal | 28930 | 146 |
| *Pelargonium × hortorum* L.H. Bailey | Malva-rosa | Ornamental | NC | 147 |
| *Pelargonium odoratissimum* (L.) L'Hér | Malva | Medicinal | 28929 | 148 |
| **Lamiaceae** |  |  |  |  |
| *Glechoma hederacea* L. | Erva-terrestre | Medicinal | 28894 | 149 |
| *Lavandula angustifolia* Mill. | Alfazema | Medicinal | 29224 | 150 |
| *Leonotis nepetifolia* (L.) R.Br. | Cordão-de-frade | Medicinal | 28977 | 151 |
| *Leonurus japonicus* Houtt. | Lavadeira | Medicinal | 29225 | 152 |
| *Melissa officinalis* L. | Melissa, cidreira | Medicinal | 28915 | 153 |
| *Mentha longifolia* (L.) L. | Levante | Medicinal/Mystical | 29227 | 154 |
| *Mentha pullegium* L. | Poejo | Medicinal | 29228 | 155 |
| *Mentha* sp. | Hortelã | Medicinal/Mystical | 28917 | 156 |
| *Mentha x villosa* Huds. | Hortelã | Edible/Medicinal | 29226 | 157 |
| *Ocimum basilicum* L. | Manjericão | Edible/Medicinal | 28922 | 158 |
| *Ocimum carnosum* (Spreng.) Link & Otto ex Benth. | Alfavaca-de-anis | Medicinal | 28923 | 159 |
| *Ocimum gratissimum* L. | Alfavaca | Medicinal | 29229 | 160 |
| *Origanum majorana* L. | Manjerona | Medicinal | 28925 | 161 |
| *Origanum vulgare* L. | Orégano | Edible/Medicinal | 29230 | 162 |
| *Plectranthus barbatus* Andrews | Boldo | Medicinal | 289402 | 163 |
| *Plectranthus scutellarioides* (L.) R.Br. | Coração-de-maria | Ornamental/Medicinal | 28941 | 164 |
| *Rosmarinus officinalis* L. | Alecrim | Medicinal/Mystical/Edible | 28951 | 165 |
| *Salvia officinalis* L. | Sálvia | Medicinal | 29231 | 166 |
| *Salvia splendens* Sellow ex Schult. | Brinco-de-negro | Ornamental | 28959 | 167 |
| *Tetradenia riparia* (Hochst.) Codd | Incenso | Medicinal/ Technological | 28990 | 168 |
| *Thymus vulgaris* L. | Tomilho | Edible/Medicinal | 28991 | 169 |
| *Vitex polygama* Cham. | Azeitona-do-mato | Fuel/Timber/Medicinal | 29232 | 170 |
| **Lauraceae** |  |  |  |  |
| *Cinnamomum verum* J.Presl. | Canela | Edible/Medicinal | 2725 | 171 |
| *Laurus nobilis* L. | Louro | Edible/Medicinal | 28908 | 172 |
| *Ocotea odorifera* (Vell.) Rohwer | Canela-sassafrás | Medicinal | 28924 | 173 |
| *Persea americana* Mill. | Abacate | Edible/Medicinal/Cosmetic | 29233 | 174 |
| **Lythraceae** |  |  |  |  |
| *Punica granatum* L. | Romã | Edible/Medicinal | 29237 | 175 |
| **Malpighiaceae** |  |  |  |  |
| *Byrsonima* A. Juss. | Muricí | Edible/Fuel/Cosmetic/Timber | 29238 | 176 |
| *Malpighia emarginata* DC. | Acerola | Edible | 29239 | 177 |
| **Malvaceae** |  |  |  |  |
| *Gossypium hirsutum* L. | Algodão | Medicinal/ Technological | 28895 | 178 |
| *Luehea divaricata* Mart. | Açoita-cavalo | Medicinal/ Technological | 28913 | 179 |
| *Sida rhombifolia* L. | Vassoura | Medicinal/ Technological | 29240 | 180 |
| *Sida urens* L. | Carrapicho-de-gancho | Medicinal | 28966 | 181 |
| **Melastomataceae** |  |  |  |  |
| *Clidemia urceolata* DC. | Cu-de-pinto | Edible/Ecological | 2726 | 182 |
| *Tibouchina estrellensis* (Raddi) Cogn. | Quaresma | Fuel/Timber | 29241 | 183 |
| **Meliaceae** |  |  |  |  |
| *Cedrella fissilis* DC. | Cedro | Timber/Medicinal/ Technological | 29242 | 184 |
| **Moraceae** |  |  |  |  |
| *Ficus* sp. | Gameleira | Technological | 29243 | 185 |
| *Ficus carica* L. | Figo | Edible/Medicinal | 29244 | 186 |
| *Morus alba* L. | Amora | Edible/Medicinal | 29245 | 187 |
| **Musaceae** |  |  |  |  |
| *Musa x paradisiaca* L. | Bananeira | Edible/Fodder/Medicinal | NC | 188 |
| **Myrtaceae** |  |  |  |  |
| *Eucalyptus* sp. | Eucalípto | Fuel/Timber | 29246 | 189 |
| *Eugenia uniflora* L. | Pitanga | Edible/Medicinal | 29247 | 190 |
| *Myrcia splendens* (Sw.) DC. | Folha-miúda | Fuel/Timber | 29248 | 191 |
| *Plinia cauliflora* (Mart.) Kausel | Jabuticaba | Edible/Medicinal | 28944 | 192 |
| *Psidium* sp. | Goiabeira | Edible/Fuel/ Technological | 29249 | 193 |
| *Psidium firmum* (O.Berg) Kausel | Araçá-amarelo | Edible | 28947 | 194 |
| *Psidium guajava* (O.Berg) Kausel | Goiaba | Edible/Fuel/Medicinal | 29250 | 195 |
| *Syzygium jambos* (L.) Alston | Jambo | Timber/Medicinal | 29251 | 196 |
| **Nyctaginaceae** |  |  |  |  |
| *Fuchsia regia* (Vell.) Munz | Bonina | Ornamental/Ecological | 28892 | 197 |
| *Mirabilis jalapa* L. | Brinco-de-princesa | Medicinal/Ornamental | 29252 | 198 |
| **Oxalidaceae** |  |  |  |  |
| *Oxalis latifolia* Kunth | Trevinho | Edible/Medicinal | 28927 | 199 |
| **Papaveraceae** |  |  |  |  |
| *Fumaria officinalis* L. | Fumária | Medicinal | 29253 | 200 |
| **Passifloraceae** |  |  |  |  |
| *Passiflora edulis* Sims | Maracujá | Edible/Medicinal | 28928 | 201 |
| *Passiflora* sp. | Maracujá-do-mato | Edible/Medicinal/Mystical | 29254 | 202 |
| **Phyllanthaceae** |  |  |  |  |
| *Phyllanthus tenellus* Roxb. | Quebra-pedra | Medicinal | 28934 | 203 |
| **Phytolaccaceae** |  |  |  |  |
| *Petiveria alliacea* L. | Guiné | Mystical/Medicinal | 28933 | 204 |
| **Pinaceae** |  |  |  |  |
| *Pinus* sp. | Pinho | Timber | 29255 | 205 |
| **Piperaceae** |  |  |  |  |
| *Piper aduncum* L. | Ruão | Medicinal/Cosmetic | 28938 | 206 |
| *Piper mollicomum* (Kunth) Kunth ex Steud. | Jaborandi | Cosmetic/Medicinal/ Technological | 29257 | 207 |
| *Piper nigrum* L. | Pimenta-do-reino | Medicinal | 29256 | 208 |
| *Piper regnellii* (Miq.) C.DC. | Chapeu-de-couro | Medicinal | 28937 | 209 |
| *Piper umbellatum* L. | Capeba | Edible/Medicinal | 28936 | 210 |
| **Plantaginaceae** |  |  |  |  |
| *Plantago major* L. | Transagem | Medicinal/Edible | 28939 | 211 |
| *Scoparia dulcis* L. | Vassourinha-doce | Medicinal/Edible | 28962 | 212 |
| **Poaceae** |  |  |  |  |
| *Coix lacryma-jobi* L. | Lágrima-de-nossa-senhora | Medicinal/Ornamental | 28877 | 213 |
| *Cymbopogon citratus* (DC.) Stapf | Erva-cidreira-de-capim | Medicinal/Edible/ | 28847 | 214 |
| *Imperata brasiliensis* Trin. | Sapé | Medicinal/ Technological | 29259 | 215 |
| *Melinis minutiflora* P.Beauv. | Capim-gordura | Fodder/Medicinal | 29258 | 216 |
| *Saccharum officinarum* L. | Cana | Edible/Medicinal | NC | 217 |
| *Zea mays* L. | Milho | Edible/Medicinal/  Technological | NC | 218 |
| **Polygalaceae** |  |  |  |  |
| *Polygala paniculata* L. | Gelol | Medicinal | 28946 | 219 |
| *Persicaria hydropiperoides* (Michx.) Small | Erva-de-bicho | Medicinal | 29260 | 220 |
| **Pontederiaceae** |  |  |  |  |
| *Eichhornia crassipes* (Mart.) Solms | Mãe-d´agua | Ornamental/ Technological | 29261 | 221 |
| **Portulacaceae** |  |  |  |  |
| *Portulaca oleracea* L. | Beldroega | Medicinal/Edible | 29262 | 222 |
| **Pteridaceae** |  |  |  |  |
| *Adiantum raddianum* C.Presl | Avenca-miúda | Medicinal/Ornamental | 28843 | 223 |
| **Rosaceae** |  |  |  |  |
| *Agrimonia eupatoria* L. | Agrimônia | Medicinal | 29263 | 224 |
| *Eryobothria japonica* (Thunb.) Lind. | Ameixa-japão | Edible/Medicinal | 28890 | 225 |
| *Malus domestica* Borkh. | Maçã | Edible/Medicinal | NC | 226 |
| *Prunus persica* (L.) Batsch | Pêssego | Edible/Medicinal | NC | 227 |
| *Rosa centifolia* L. | Rosa-de-remédio | Medicinal/Ornamental | 29264 | 228 |
| *Rubus erythrocladus* Mart. ex Hook.f. | Framboesa | Edible/Medicinal | 28955 | 229 |
| *Rubus idaeus* L. | Amora-branca | Edible/Medicinal | 28954 | 230 |
| *Rubus rosifolius* Sm. | Moranguinho | Edible/Medicinal | 28957 | 231 |
| *Rubus sellowii* Cham. & Schltdl. | Cereja | Edible/ Technological | NC | 232 |
| *Spiraea cantoniensis* Lour. | Coroa-de-noiva | Ornamental | 28978 | 233 |
| **Rubiaceae** |  |  |  |  |
| *Bathysa australis* (A.St.-Hil.) K.Schum. | Quina | Medicinal | 28862 | 234 |
| *Borreria verticillata* (L.) G.Mey. | Mercúrio-vegetal | Technological | 29265 | 235 |
| *Coffea arabica* L. | Café | Edible/Medicinal | NC | 236 |
| *Cordiera elliptica* (Cham.) Kuntze | Marmelinho | Medicinal/Edible | 29266 | 237 |
| *Remijia ferruginea* (A.St.-Hil.) K.Schum. | Quina-do-cerrado | Medicinal | 29324 | 238 |
| **Rutaceae** |  |  |  |  |
| *Citrus limon* (L.) Osbeck | Limão | Edible/Medicinal/ Technological | NC | 239 |
| *Citrus medica* L. | Cidra | Edible/Cosmetic/Medicinal | NC | 240 |
| *Citrus reticulata* Blanco | Mexerica-candongueira | Edible/Medicinal | NC | 241 |
| *Citrus x aurantium* L. | Laranja | Edible/Medicinal | NC | 242 |
| *Ruta graveolens* L. | Arruda | Mystical/Medicinal | 28958 | 243 |
| *Zanthoxylum rhoifolium* Lam. | Maminha-de-porca | Medicinal | 2727 | 244 |
| **Sapindaceae** |  |  |  |  |
| *Cupania vernalis* Cambess. | Camboatá | Fuel | 29268 | 245 |
| **Sapotaceae** |  |  |  |  |
| *Manilkara* sp. | Parajú | Timber | NC | 246 |
| **Scrophulariaceae** |  |  |  |  |
| *Buddleja stachyoides* Cham. & Schltdl. | Barbaço | Medicinal | 28867 | 247 |
| **Siparunaceae** |  |  |  |  |
| *Siparuna brasiliensis* (Spreng.) A.DC. | Folha-santa, negramina | Medicinal/Mystical | 29269 | 248 |
| *Smilax aspera* L. | Salsaparrilha | Medicinal | 29270 | 249 |
| **Solanaceae** |  |  |  |  |
| *Acnistus arborescens* (L.) Schltdl. | Mariana | Ecological/Edible | 28841 | 250 |
| *Brugmansia suaveolens* (Humb. & Bonpl. ex Willd.) Bercht. & J.Presl | Babado | Medicinal/Ornamental | 29271 | 251 |
| *Capsicum* sp. | Pimenta | Edible | 29275 | 252 |
| *Cestrum nocturnum* L. | Dama-da-noite | Ornamental | 28874 | 253 |
| *Lycopersicum* sp. | Tomatinho | Edible | 29272 | 254 |
| *Nicotiana tabacum* L. | Fumo | Medicinal/ Technological | 29273 | 255 |
| *Physalis angulata* L. | Joá-de-capote | Edible | 29274 | 256 |
| *Solanum aethiopicum* L. | Jiló | Edible/Medicinal | 29276 | 257 |
| *Solanum alternatopinnatum* Steud. | Jequeri | Edible/Medicinal | 28974 | 258 |
| *Solanum americanum* Mill. | Maria-preta | Edible/ Technological/Medicinal | 28975 | 259 |
| *Solanum betaceum* Mill. | Tomate-mango | Edible | 29277 | 260 |
| *Solanum capsicoides* All. | Joá | Edible/Medicinal | 28969 | 261 |
| *Solanum cernuum* Vell. | Barba-de-bode, costa-branca, panacéia | Medicinal | 28967 | 262 |
| *Solanum lycocarpum* Rooyen ex L. | Lobeira | Medicinal | 29278 | 263 |
| *Solanum melongena* L. | Berinjela | Edible/Medicinal | NC | 264 |
| *Solanum muricatum* Ait. | Melão-andino | Edible | 28973 | 265 |
| *Solanum tuberosum* L. | Batata | Edible | NC | 266 |
| **Theaceae** |  |  |  |  |
| *Camellia sinensis* (L.) Kuntze | Chá-preto | Edible/Medicinal | 28870 | 267 |
| **Tropaeolaceae** |  |  |  |  |
| *Tropaeolum majus* L. | Chaguinha, capuchinha | Edible/Medicinal | 2728 | 268 |
| **Typhaceae** |  |  |  |  |
| *Typha domingensis* Pers. | Taboa | Technological | 2729 | 269 |
| **Urticaceae** |  |  |  |  |
| *Boehmeria caudata* Sw. | Urtiga-escura | Edible/Medicinal/Fodder | 2698 | 270 |
| *Cecropia hololeuca* Miq. | Embaúba-branca | Fuel/Medicinal/ Technological | 29282 | 271 |
| *Cecropia pachystachia* Trécul | Embaúba | Timber/Medicinal/ Technological | 29283 | 272 |
| *Parietaria officinalis* L. | Paletária | Medicinal | 29285 | 273 |
| *Urera baccifera* (L.) Gaudich. ex Wedd. | Cansansão | Medicinal | 29284 | 274 |
| *Urtica urens* L. | Urtiga | Edible/Medicinal | 28992 | 275 |
| **Velloziaceae** |  |  |  |  |
| *Vellozia compacta* Mart. | Canela-de-ema | Medicinal/Fuel | 2707 | 276 |
| **Verbenaceae** |  |  |  |  |
| *Aloysia pulchra* (Briq.) Moldenke | Boldo-chileno | Medicinal | 28846 | 277 |
| *Duranta erecta* L. | Chuva-de-ouro | Ornamental | 29286 | 278 |
| *Lantana camara* L. | Camarazinho | Medicinal | 28905 | 279 |
| *Lantana fucata* Lindl. | Bem-me-quer | Medicinal | 28906 | 280 |
| *Lippia alba* (Mill.) N.E.Br. ex Britton & P.Wilson | Cidreira, Melissa | Medicinal | 28910 | 281 |
| *Stachytarpheta cayennensis* (Rich.) Vahl | Jurubão | Medicinal | 28979 | 282 |
| **Violaceae** |  |  |  |  |
| *Anchietea pyrifolia* (Mart.) G.Don | Suma-branca | Medicinal | 28849 | 283 |
| *Viola odorata* L. | Violina | Medicinal/Ornamental | 28994 | 284 |
| **Vitaceae** |  |  |  |  |
| *Cissus verticillata* (L.) Nicolson & C.E.Jarvis | Insulina | Medicinal | 29289 | 285 |
| *Vitis vinifera* L. | Uva | Edible/Medicinal | 29287 | 286 |
| **Xanthorrhoeaceae** |  |  |  |  |
| *Aloe vera* (L.) Burm.f. | Babosa | Medicinal/Cosmetic | 29288 | 287 |
| **Zingiberaceae** |  |  |  |  |
| *Curcuma longa* L. | Açafrão | Edible/ Technological | 29291 | 288 |
| *Hedychium coronarium* J.Koenig | Lirio-do-campo | Medicinal/Ecological | 29292 | 289 |
| *Renealmia alpinia*(Rottb.) Maas | Pacová | Medicinal | 2723 | 290 |
| *Zingiber officinale* Roscou | Gengibre | Edible/Medicinal | 28996 | 291 |
|  | | | | |
