# Supplementary material for: Gender differences in traditional knowledge of useful plants in a Brazilian community
Source: PLoS One. 2021 Jul 28;16(7):e0253820. doi: 10.1371/journal.pone.0253820 (PMC8318302; doi:10.1371/journal.pone.0253820)
Supplement: S2 Table — *Indicate metrics significance against Patefield null model; St. = Standardized values for each metric considering the number of standard deviations above the average value recorded in 999 randomizations. Standardized values lower or greater than two indicate significant values, since they represent how many standard deviations the real observed metric is far from the mean of 999 values generated from randomized networks. Therefore, instead of P-values, we used standardized values to estimate metrics’ significance. (DOCX) [file pone.0253820.s002.docx]

**S2 Table.**  Comparison of connectance (C), niche overlap in resource use (Horn), modularity (Q), and their respective significances (N= 999 randomizations) for woman and man ethnobotanical networks from an urban community, Ouro Preto, Brazil (*indicate metrics significance against Patefield null model; St. = Standardized values for each metric considering the number of standard deviations above the average value recorded in 999 randomizations. Standardized values lower or greater than two indicate significant values, since they represent how many standard deviations the real observed metric is far from the mean of 999 values generated from randomized networks. Therefore, instead of P-values, we used standardized values to estimate metrics’ significance.

| Gender | C | St. C | Horn | St. Horn | Q | St. Q |
| --- | --- | --- | --- | --- | --- | --- |
| Women | 0.17 | 16.07* | 0.21 | 19.29* | 0.27 | 3.42* |
| Men | 0.07 | 7.34* | 0.07 | -0.16 | 0.40 | -3.62* |
